# Progress in treatment of small-cell lung cancer: role of CPT-11

**DOI:** 10.1038/sj.bjc.6601456

**Published:** 2003-12-09

**Authors:** N Saijo

**Affiliations:** 1 Internal Medicine and Thoracic Oncology Division, National Cancer Center Hospital, Tuskiji 5-1-1, Chuo-ku, Tokyo 104-0045, Japan

**Keywords:** Small-cell lung cancer, JCOG, CPT-11

## Abstract

Small-cell lung cancer (SCLC) accounts for approximately 15% of all cases of lung cancer and is a particularly aggressive form of lung cancer characterised by a poor prognosis, rapid tumour growth, and early metastasis. Roughly, two-thirds of patients with SCLC present with extensive disease (ED) and one-third with limited disease (LD). Combination chemotherapy is the most effective treatment modality for SCLC, and several new agents, including carboplatin, ifosfamide, taxans, and topotecan, have been demonstrated to be active; however, there are no data on the survival benefit of these drugs. A CPT-11+ cisplatin regimen has shown improvement in overall survival over the global gold standard regimen, etoposide + cisplatin (Japanese Clinical Oncology Group: JCOG 9511), and three confirmatory randomised controlled trials are in progress to determine the reproducibility of the JCOG 9511 study. JCOG is evaluating the role of CPT-11 and a new triplet regimen containing CPT-11 in limited-stage SCLC. Strategies and the current protocols of the JCOG are presented and discussed. In the future, it will be essential to evaluate molecular target-based drugs for LD and ED SCLC with new standard combination chemotherapy regimens that include CPT-11.

Lung cancer is one of the most common cancers not only in Western countries but also in Japan as well. Small-cell lung cancer (SCLC) has a poor prognosis and accounts for approximately 15% of all lung cancer deaths. It is characterised by an aggressive clinical course, rapid tumour growth, and metastatic spread, and despite being a chemo- and radiosensitive tumour, majority of SCLC patients die because of recurrence after the response to initial treatment. Roughly, one-third present with limited-stage disease (LD), and the remainder with extensive-stage disease (ED). Surgery and radiation therapy have been used as a single modality or in combined modality therapy but have resulted in few cures (Hanna and Einhorn 2002). The standard treatment strategy for LD-SCLC and ED-SCLC is chemoradiotherapy and chemotherapy, respectively (Evans *et al*, 1987; Fukuoka *et al*, 1991; Fukuda *et al*, 1996; Saijo 2002; Sandler 2002). A cisplatin (CDDP) + etoposide (VP-16) (PE) regimen is the most frequently used chemotherapeutic regimen worldwide, and various strategies, such as the introduction of new anticancer drugs, non-cross-resistant alternative chemotherapy (Evans *et al*, 1987; Fukuoka *et al*, 1991; Murray *et al*, 2001), weekly dose-intensive chemotherapy (Murray *et al*, 1991), high-dose chemotherapy with haematopoietic stem cell support (Elias *et al*, 1993), and the evaluation of timing, dose, or fractionation of radiation therapy have been tested in randomised controlled trials to improve the results of treatment of SCLC (Sandler, 2001). Several new anticancer drugs, including ifosfamide and taxans, have been shown to be active against SCLC, but, have not resulted in improvement of overall survival. The most active one of these drugs to date has been the topoisomerase I inhibitor irinotecan (CPT-11) (Saijo *et al*, 1994; Le Chevalier *et al*, 1997; Devore *et al*, 1998; Kelly, 2001; Shihabi and Belani, 2001). This review focuses on the contribution of CPT-11 to improvement of the results of treatment of SCLC and discusses the future development of CPT-11 in the treatment of SCLC.

## SINGLE-AGENT ACTIVITY OF CPT-11 AGAINST SCLC

In the phase I trial of CPT-11, the recommended dose of CPT-11 was determined to be 100–125 mg m^−2^ once a week and 150 mg m^−2^ every 2 weeks (Negoro *et al*, 1991a,1991b).

A multi-institutional phase II study was conducted in previously treated and untreated SCLC patients, and nine out of 27 previously treated patients and four out of 8 previously untreated patients experienced an objective tumour response (Kanzawa *et al*, 1990; Negoro *et al*, 1991a,1991b). Masuda used CPT-11 100 mg/m^−2^ as a 90-min infusion every week to treat 16 patients with refractory or relapsed SCLC, and seven out of 15 evaluable patients responded to CPT-11 (Masuda *et al*, 1992).

In addition to SCLC, CPT-11 has been demonstrated to be active as single-agent chemotherapy for non-small-cell-lung-cancer, colon cancer, breast cancer, uterine cancer, stomach cancer, ovarian cancer, etc.

## DEVELOPMENT OF THE CPT-11+CDDP REGIMEN

It is essential to combine the maximum effective doses of active anticancer drugs in order to obtain a better therapeutic effect. We have demonstrated that combinations of cisplatin with topoisomerase I inhibitors have an exclusively synergistic effect (Kanzawa *et al*, 1990; Fukuda *et al*, 1996). DNA interstrand crosslinks are significantly increased by such combinations, and DNA repair after interstrand crosslinks formed by cisplatin is inhibited by topoisomerase inhibitors. On the other hand, the topoisomerase I inhibitory activities of CPT-11 and SN-38 are increased by CDDP. In nude mice transplanted with human SCLC, the CPT-11 and CDDP combination showed a synergistic effect (Tamura *et al*, 1997). Phase I studies of CPT-11 and CDDP combinations with or without the support of granulocyte-colony stimulating factor (G-CSF) were conducted in untreated patients with stage IV NSCLC. CPT-11 was given on days 1, 8, and 15, and CDDP on day 1, and the schedule was repeated every 4 weeks. The dose of CDDP was fixed at 60 or 80 mg m^−2^. Without the support of G-CSF, the recommended dose of CPT-11 for a phase II study in combination with 60 and 80 mg m^−2^ of CDDP was 80 and 60 mg m^−2^, respectively. The dose-limiting toxicities were granulocytopenia and diarrhoea (Masuda *et al*, 1993).

## PHASE II STUDY OF CPT-11 AND CDDP

The West Japan Thoracic Oncology Group (WJTOG) conducted a phase II trial of CPT-11 and CDDP in SCLC. In all 75 untreated patients with SCLC were treated with 80 or 60 mg m^−2^ of CPT-11 on days 1, 8, and 15 and 60 mg m^−2^ of CDDP on day 1 every 28 days. Patients with LD-SCLC received thoracic irradiation after four cycles of chemotherapy whereas patients with ED received four or six cycles of chemotherapy. The dose of CPT-11 was reduced to 60 mg m^−2^ after four of 10 patients experienced severe haematological toxicity, diarrhoea, and hepatic toxicity, while one patient died of toxicity. Among the 35 ED patients, the objective response rate was 86% (30 patients), with 10 patients (29%) having a complete response (CR). The median survival time was 13 months. Among the 40 LD patients, 33 (83%) had a response and 12 (30%) had a CR, and the median survival time for this subset of patients was 14.3% months. The significant grade 3/4 toxicities were neutropenia (77%), anaemia (39%), nausea (35%), and diarrhoea (19%) (Kudoh *et al*, 1998). These results were very encouraging, especially for ED-SCLC.

## PHASE III STUDY OF CPT-11+CDDP *VS* VP-16+CDDP (JCOG 9511)

Based on the results of a study of the CPT-11+CDDP combination, JCOG conducted a phase III study comparing CPT-11+CDDP with VP-16+CDDP for untreated ED-SCLC.

The study arm consisted of CPT-11 60 mg m^−2^ on days 1, 8, and 15 and CDDP 60 mg m^−2^ on day 1, every four weeks, for four courses. The control arm consisted of VP-16 100 mg m^−2^ on days 1–3 and CDDP 80 mg m^−2^ on day 1, every 3 weeks, for four courses. The initial sample size was 230 patients, 115 cases per arm, in order to demonstrate a 30% increase in overall survival with the significant difference of *α* error *P*<0.05 and *β* error <0.8. However, enrollment into the study was stopped because of a huge difference (*P*=0.00025) in survival on an interim analysis.

**Figure 1:**
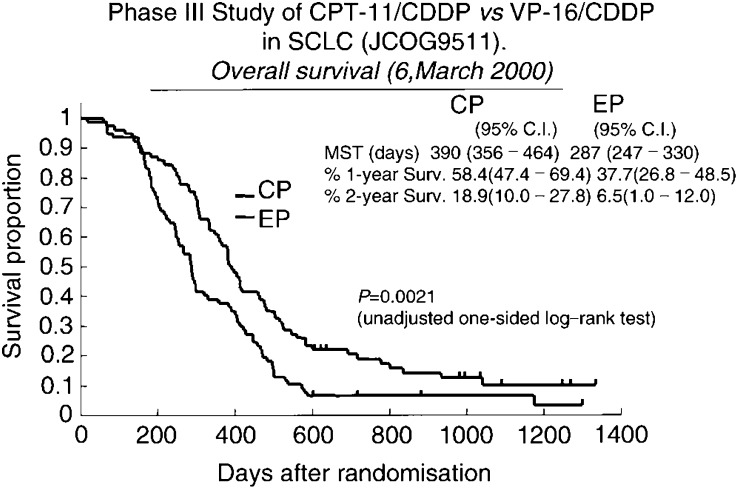
Phase III Study of CPT-11/CDD *vs* VP-16/CDDP in SCLC (JCOG9511).

In the final data, 154 patients were randomised, 77 into each arm, and the overall and CR rates were 84 and 3%, respectively, in the CPT-11+ CDDP arm and 68 and 9%, respectively, in the VP-16+CDDP arm, (*P*=0.02 for the difference in overall response rate). The median survival time was 12.8 and 9.4 months in the CPT-11+CDDP arm and VP-16+CDDP arm, respectively. The CPT-11 + CDDP arm was characterised by significantly better survival than the standard regimen, VP-16+ CDDP (*P*=0.002 unadjusted one-sided log-rank test). The rates (95% confidence limit) of overall survival in CPT-11+CDDP arm were 58.4 (47.7–69.4) % at 1 year and 19.5 (10.0–27.8)% at 2 years: in VP-16 plus CDDP arm the rates of overall survival at these time points, were 37.7 (26.8–48.5) % and 5.2 (1.0-12.0)%, respectively (Figure 1

).

Grade 3/4 leukopenia occurred in 27% of patients on CPT-11 + CDDP and in 52% on VP-16 + CDDP (*P*=0.002). Grade 3/4 neutropenia developed in 65% of patients on CPT-11+CDDP and in 92% on VP-16+CDDP (*P*<0.001). Grade 3/4 diarrhoea, on the other hand, occurred in 16% of the patients in the CPT-11+CDDP arm, but in none of the patients in the VP-16+ CDDP arm (*P*<0.001). Treatment with four cycles of CPT-11+ CDDP every 4 weeks yielded a highly significant improvement in survival in ED-SCLC over standard VP-16+ CDDP, with less myelosuppression. There were no statistically significant differences between patient characteristics, and there were slightly more patients with more favourable pretreatment characteristics, such as female gender, PS 0 or 1, fewer patients with brain metastasis, in the CPT-11+ CDDP arm. In view of the small sample size in the study, even small differences in patient characteristics may have resulted in outcome differences between the two arms. Another problem was compliance with the regimen. Only 29% of patients received the full dose of chemotherapy without skips or delays in CPT-11+ CDDP arm. Based on the results of JCOG 9511, CPT-11+ CDDP is considered the reference chemotherapy arm for ED-SCLC in future JCOG trials (Noda *et al*, 2002).

The data of JCOG 9511 are clearly interesting, however, only 154 patients have been randomised. It is essential to evaluate the reproducibility of the effect of CPT-11. Three randomised controlled trials, PHA GMA 262, SWOG SO124, and Aventis GMA 3001, are in progress; to confirm the data obtained in JCOG 9511 (Table 1

**Table 1:** SCLC studies designed to support registration and their current situation

**CPT-11+cisplatin small cell-lung cancer studies: study regimens sample size, and survival assumptions**									
	**Test regimen**			**Control regimen**			**Patients**	**Survival**	
**Study**	**CPT-11**	**Cisplatin**	**Cycles**	**Etoposide**	**Cisplatin**	**Cycles**	**Test/control (Accural time)**	**Hazard ratio**	**Status**
JCOG 9511	60 mg m^−2^ D1,8,15	60 mg m^−2^ D1	Q4 weeks × 4 cycles	100 mg m^−2^ D1–3	80 mg m^−2^ D1	Q 3 weeks × 4 cycles	77/77	1.44	Completed
PHA GMA 262 (North American study)	65 mg m^−2^ D1,8	30 mg m^−2^ D1,8	Q 3 weeks × ⩾4 cycles	120 mg m^−2^ D1,3	60 mg m^−2^ D1	Q 3 weeks × ⩾4 cycles	200/100(28 months)	1.33	2 : 1 randomisation-PS2 pts excluded as of 1Q02 145 pts accured to date (sites in US, Canada, and Australia)
Swog S0124	60 mg m^−2^ D1,8,15	60 mg m^−2^ D1	Q 4 weeks × ⩾4 cycles	100 mg m^−2^ D1–3	80 mg m^−2^ D1	Q 3 weeks × ⩾4 cycles	310/310 (48 months)	1.33	PS2 pts excluded SWOG kick-off meeting held 26 April, 2002
Aventis GMA 3001	60 mg m^−2^ D1,8	80 mg m^−2^ D1	Q 3 weeks × ⩾6 cycles	100 mg m^−2^D1–3	80 mg m^−2^ D1	Q 3 weeks ⩾4 cycles	164/164 (18 months)	1.44	First pt. anticipated in June 2000. In all, 13 participating countries (EU and Asia Pacific)

).

## CHEMOTHERAPY WITH CISPLATIN+CPT-11+VP-16

The VP-16+CDDP regimen is still considered the standard regimen in North America, and CPT-11+CDDP showed a survival benefit in JCOG9511. The combination chemotherapy with CPT-11+ VP-16 also shows promising results (Karato *et al*, 1993; Masuda *et al*, 1998). It is quite reasonable to investigate the three-drug combination CPT-11+VP-16+CDDP to increase efficacy without increasing toxicity (Table 2

**Table 2:** Recent trials of cisplatin/irinotecan/etoposide for SCLC in Japan

**Trial**	**Patients**	**Treatment**
JCOG9902-DI (Randomised phase II)	Extensive disease (*n*=30)	Cisplatin 25 mg m^−2^ day 1
		Irinotecan 90 mg m^−2^ day 1
		Etoposide 60 mg m^−2^ days 1–3
		*versus* Cisplatin 60 mg m^−2^ day 1
JCOG9903-DI (pilot study)	Limited disease (*n*=30)	Cycle 1: Cisplatin 80 mg m^−2^ day 1
		Etoposide 100 mg /m^−2^ 1–3 TRT b.i.d. to 45 Gy
Multicentre (Phase II)	Sensitive relapse (*n*=33)	Cisplatin 25 mg m^−2^ day 1
	Refractory (*n*=27)	Irinotecan 90 mg m^−2^ day 1
		Etoposide 60 mg m^−2^ days 1–3, weeks 1,3, 5, 7, 9 weekly

). The JCOG has conducted two phase I/II studies of CDDP + VP-16+CPT-11 combination chemotherapy using two different schedules: weekly (JCOG 9507) and four-weekly (JCOG 9512) (Ohe and Saijo, 2002). Based on the doses determined in the two phase I studies, a randomized phase II study (JCOG 9902-DI) Table 3

**Table 3:** Randomised phase II (JCOG 9902-DI) study of CDDP, CPT-11, and VP-16 administered weekly or every 4 weeks for ED-SCLC (August 1999–April 2001)

	**Arm A (Weekly)**	**Arm B (q 4 weeks)**
No of patients	30	30
Median dose intensity (CDDP/CPT-11/VP-16) mg m^−2^ week^−1^	21/40/68	15/35/37
Median total dose (CDDP/CPT-11/VP-16) mg m^−2^	225/450/720	240/563/600
CR/PR	2/23	5/18
SD/PD/NE	1/3/1	0/4/3
RR (%)	83	77
Gr toxicity (%) leucopenia/neutropenia	50/57	53/87
MST (months)	8.9	12.9
1-Year survival (%)	40	56

was conducted comparing weekly and every-four-week schedules of this three-drug combination to select the appropriate arm for the future phase III trial (JCOG 9902-DI) (Figure 2

**Figure 2:**
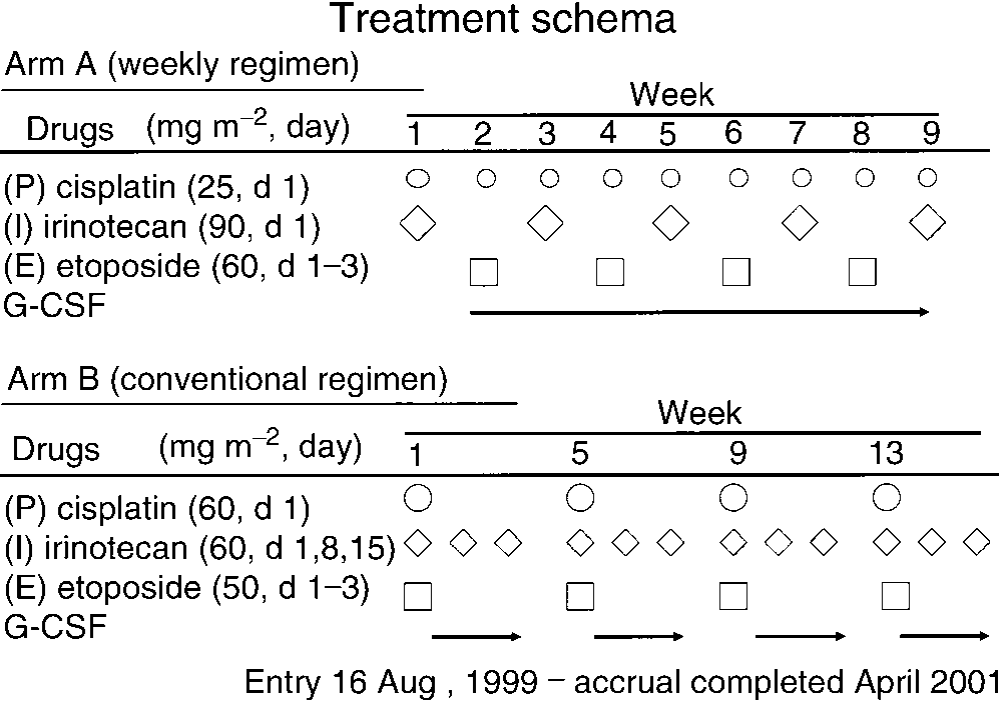
Treatment schema.

).

Weekly chemotherapy consisted of CDDP 25 mg m^−2^ on day 1 of weeks 1–9, CPT-11 90 mg m^−2^ on day 1 of weeks 1, 3, 5, 7, and 9, and VP-16 60 mg m^−2^ on days 1–3 of weeks 2, 4, 6, and 8, G-CSF support. Chemotherapy for the every-4-week schedule consists of CDDP 60 mg/m^2^ on day 1, CPT-11 60 mg/m^2^ on days, 1, 8, and 15, and VP-16 50 mg m^−2^ on days 1–3, with G-CSF support. The doses of these agents were decided in phase I studies, but the schedule for the weekly regimen has been modified. The original JCOG 9507 schedule consisted of CDDP 25 mg m^−2^ on day 1 of weeks 1–9, escalating doses of CPT-11 on day 1 of weeks 2, 4, 6, and 8, and VP-16 60 mg m^−2^ on days 1–3 of weeks 1, 3, 5, 7, and 9, with G-CSF support. Since we concluded that irinotecan is more active against SCLC than VP-16 based on the results of JCOG 9511, CPT-11 and CDDP is given in week 1 instead of VP-16 and CDDP in JCOG 9902-DI.

A total of 60 patients with chemotherapy-naïve ED-SCLC have been enrolled in the study, and the eligibility criteria included age ⩽70 years, PS 0-2, and no prior therapy. Arm A consisted of P (25 mg m^−2^ day 1) weekly for 9 weeks, CPT-11 (90 day 1) in weeks 1, 3, 5, 7 and 9, and VP-16 (60 days 1–3) in weeks 2, 4, 6, and 8. Arm B consisted of cisplatin (60 mg m^−2^ day 1), I (60 days 1, 8, 15), and VP-16 (50 days 1–3) every 4 weeks for four cycles. G-CSF support was used in both arms. Between August 1999 and October 2000, 60 patients were randomised to arm A (*n*=30) and arm B (*n*=30).

Full cycles were delivered in 73 and 70% of the patients in arms A and B, respectively. The incidences of grade 3–4 neutropenia, anaemia, thrombocytopenia, infection, and diarrhoea were 57, 43, 27, 7, and 7%, respectively, in arm A, and 87, 47, 10, 13, and 10%, respectively in arm B. Treatment-related death was observed in one patient in arm A. The CR rate and partial response (PR) rate were 7 and 77%, respectively, in arm A, and 17 and 60% in arm B. MST and the 1-year survival rate were 8.9 months and 40%, respectively, in arm A, and 12.8 months and 56% in arm B. In conclusion, arm B was appropriate as an investigational arm in further phase III studies (Sekine *et al*, 2003). JCOG is conducting a randomised controlled trial comparing a CPT-11+CDDP regimen consisting of CPT-11 60 mg m^−2^ on days 1 and 8, and CDDP 60 mg m^−2^ on day 1 with a CPT-11 + VP-16 + CDDP regimen consisting of CPT-11 60 mg m^−2^ on days 1 and 8, CDDP 60 mg m^−2^ on day 1, and VP-16 50 mg m^−2^ on days 1–3. Both the regimens will be given every 3 weeks.

## TREATMENT OF RECURRENT SCLC WITH A CPT-11+CDDP+VP-16 REGIMEN

There is no standard treatment for relapsed SCLC (Schulthesis *et al*, 2001). Since CPT-11 and VP-16 have been studied in several phase II trials as active agents for relapsed SCLC, we conducted a phase II study to evaluate the efficacy and toxicity of CPT-11 combined with weekly CDDP and VP-16 (PE) in patients (pts) with SCLC who had relapsed at least 8 weeks after completion of first-line therapy. Patients had to have measurable or assessable disease, be age 75 years old or under, and have performance status of 0 to 2 (ECOG), and adequate organ function. The PE/CPT regimen consisted of CDDP 25 mg m^−2^ on day 1 at 1-week intervals for 9 weeks (at least 6 weeks), VP-16 60 mg m^−2^ on days 1–3 in weeks 1, 3, 5, 7, and 9 (at least in weeks 1, 3, 5), and CPT-11 90 mg m^−2^ in weeks 2, 4, 6, and 8 (at least in weeks 2, 4, 6). After day 1 in week 2, G-CSF was administered on days when no cytotoxic drugs were given.

Between October 1998 and March 2001, 40 pts were enrolled in this study. Patient characteristics were median age 67 years (range 41–74), 29 males and 11 females, 5 LD and 35 ED. Prior chemotherapy included PE in 11 patients, carboplatin and VP-16 in 11, PE/CODE in six, cisplatin and irinotecan in six, PE/CPT in two, and others in four patients. Eight patients received thoracic radiotherapy. In all, 32 patients (80%) completed six or more weeks of chemotherapy. There were five CRs and 26 PRs, and the overall response rate was 78% (95% CI 64.6–90.4%). The median survival time was 11.4 months. Grade 3/4 neutropenia and thrombocytopenia were observed in 73 and 33%, respectively. Grade 3 nausea/vomiting was observed in 8%, and grade 3/4 diarrohea in 8%. In conclusion, the PE/CPT regimen was active against relapsed SCLC and well tolerated (Goto *et al*, 2002).

## CPT-11-CONTAINING REGIMENS FOR LD-SCLC

CPT-11-containing regimens are considered to be one of the most promising strategies for improving the survival of LD-SCLC patients. Early concurrent thoracic radiation therapy with combination chemotherapy consisting of VP-16/CDDP is a standard treatment for LD-SCLC patients (Perry *et al* 1987; Pignon *et al*, 1992; Murray *et al*, 1993; Jeremic *et al*, 1997; Work *et al*, 1997). Twice-daily thoracic radiation therapy and prophylactic cranial irradiation after a CR have recently been added to the standard treatment (Trussi *et al*, 1999; Takada *et al*, 2002). Thus, concurrent twice-daily thoracic radiation therapy with combination chemotherapy consisting of CPT-11/CDDP may be the most powerful treatment for LD-SCLC patients, if the full dose of CPT-11 can be given with acceptable toxicity. Previously, the JCOG conducted a dose-finding study for CPT-11/CDDP plus concurrent radiation therapy in unresectable stage III NSCLC (JCOG 9405). The CPT-11 dose intensity in that study was low because of the need to skip CPT-11 administration of days 8 and/or 15 as a result of leukopenia or diarrhoea, and the radiation therapy completion rate was low as well. Based on the results of JCOG 9405, chemotherapy with CPT-11/CDDP plus concurrent radiation therapy was deemed unacceptable (Yokoyama *et al*, 1998).

The JCOG also conducted a phase II study of single-agent CPT-11 (60 mg m^−2^) in combination with radiation therapy in NSCLC (JCOG 9504). When CPT-11 is used as a single agent with concurrent radiation therapy, the dose of CPT-11 must be reduced from 100 to 60 mg m^−2^ in a weekly schedule. However, this dose reduction may reduce the efficacy of CPT-11 in the treatment of LD-SCLC patients. Full-dose chemotherapy consisting of VP-16/CDDP can also be used in combination with concurrent radiation therapy (Table 3).

Japanese Clinical Oncology Group, therefore, conducted a pilot study of concurrent twice-daily thoracic radiation therapy plus VP-16/CDDP followed by three cycles of chemotherapy with standard doses of CPT-11/CDDP (JCOG-9903-DI). To be eligible, patients had to have previously untreated LD-SCLC, be <75 years of age, and have an ECOG performance status (PS) of 0-2 and adequate organ function. Treatment consisted of 80 mg m^−2^ of CDDP on day 1 and 100 mg m^−2^ of VP-16 on day 1–3. Accelerated twice daily thoracic radiation therapy (TRT) started on day 2 and involved administration of 1.5 Gy in 30 fractions over a period of 3 weeks. After completion of PE/TRT, the patients received 3 cycles of CPT-11+CDDP started on day 29. IP consisted of 60 mg m^−2^ of CPT-11 on days 1, 8, and 15 and 60 mg m^−2^ of CDDP on day 1 every 4 weeks.

Between October 1999 and July 2000, 31 patients were enrolled in the study. Patient characteristics were: male 27, female 4, median age 62 years, range 43–74 years, PS 0/1: 8/23. Of the 30 patients who received the protocol treatment, 11 pts had a CR, 18 had a PR, and one progressive disease, for a response rate of 97%. Totally 25 patients received CPT-11+CDDP. Grade 3/4 toxicities during CPT-11+CDDP phase were: WBC (48/12%), plt (4/0%), Hb (44/—), diarrohea (4/4%). No treatment-related deaths were observed. The 1-year survival rate was 79.3% among patients who received the protocol treatment, and 87.5% among pts who received the CPT-11+CDDP regimen. The median survival time has not been obtained (Table 4

**Table 4:** Phase II (JCOG 9903-DI) study of CDDP and VP-16 plus concurrent AHTRT followed by three cycles of CPT-11 and CDDP in LD-SCLC (October 1999–July 2000)

**No of patients**		**31 (male 27, female four)**
Delivery of treatment	EP/TRT	30
Cycles of IP	1/2/3	24/22/17
	(Causes of treatment off: toxicity and patient refusal)	
CR/PR		12/17
SD/PD/not treated		0/1/1
RR (%)		94
Toxicity of PE/TRT	Grade 4	14/19 (47/63%)
	Leukopenia/neutropenia	
Toxicity of IP	Grade 4	5/5 (21/21%)
	Leukopenia/neutropenia	
MST/-year survival		Not available/79.3%

) (Mori *et al*, 2002). In conclusion, PE plus concurrent twice daily TRT followed by three cycles of CPT-11+CDDP is a safe and active regimen with an encouraging 1-year survival rate. JCOG is conducting a phase III study comparing it with the standard PE concurrent accerelated hyperfractionated TRT regimen (Figure 3

**Figure 3:**
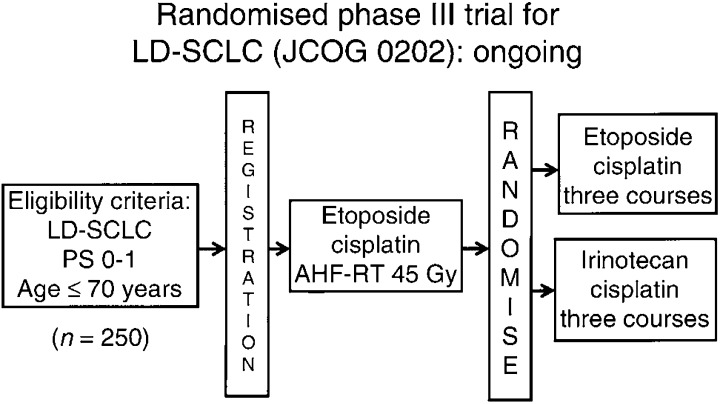
Randomised phase III trial for LD-SCLC (JCOG 02002): Ongoing.

).

## CONCLUSIONS

Although dramatic improvements in survival in response to combination chemotherapy were observed in the 1970s and 1980s, the results of treating SCLC had reached a plateau. Novel drugs and approaches are essential to further improve the outcome of treatment. Significant activity of CPT-11 in the treatment of patients with SCLC provides the first evidence in 20 years that the survival of SCLC patients can be prolonged, although we must await the results of confirmatory studies. Current and future trials of CPT-11 will be aimed at evaluating three-drug regimens containing CDDP and CPT-11 in multimodality therapies and targeting limited-stage disease. In addition, CPT-11 may have the potential to be a part of second-line treatment for SCLC. In the future, combining a CPT-11 containing regimen with target-based therapy will also be a major focus of new directions in SCLC therapy in order to overcome resistance to CPT-11. In Japan, amrubicin has been demonstrated to be extremely active against SCLC and the optimal dose for the combination of amrubicin and CDDP has been decided. The role of amrubicin for the treatment of SCLC should also be investigated.
